# Accurate Calibration of Multi-LiDAR-Multi-Camera Systems

**DOI:** 10.3390/s18072139

**Published:** 2018-07-03

**Authors:** Zoltán Pusztai, Iván Eichhardt, Levente Hajder

**Affiliations:** 1 Geometric Computer Vision Group, Machine Perception Laboratory, MTA SZTAKI, Kende st. 17, 1111 Budapest, Hungary; iffanka@gmail.com (I.E.); hajder@inf.elte.hu (L.H.); 2 Department of Algorithms and Their Applications, Eötvös Loránd University, Pázmány Péter stny. 1/C., 1117 Budapest, Hungary

**Keywords:** LiDAR, camera, LiDAR camera system, machine perception, extrinsic calibration, autonomous driving

## Abstract

As autonomous driving attracts more and more attention these days, the algorithms and sensors used for machine perception become popular in research, as well. This paper investigates the extrinsic calibration of two frequently-applied sensors: the camera and Light Detection and Ranging (LiDAR). The calibration can be done with the help of ordinary boxes. It contains an iterative refinement step, which is proven to converge to the box in the LiDAR point cloud, and can be used for system calibration containing multiple LiDARs and cameras. For that purpose, a bundle adjustment-like minimization is also presented. The accuracy of the method is evaluated on both synthetic and real-world data, outperforming the state-of-the-art techniques. The method is general in the sense that it is both LiDAR and camera-type independent, and only the intrinsic camera parameters have to be known. Finally, a method for determining the 2D bounding box of the car chassis from LiDAR point clouds is also presented in order to determine the car body border with respect to the calibrated sensors.

## 1. Introduction

Nowadays, autonomous driving is in the focus of several research communities and industrial partners. Mapping of the surroundings is a basic task in machine perception. For that task, multiple modalities are used, e.g., cameras, LiDARs (Light Detection and Ranging), RADARs, IMU, GPS, etc. In order to work these instruments together, their relative position and orientation need to be known a priori; thus, extrinsic sensor calibration is needed. This paper introduces a new method for camera and LiDAR system extrinsic calibration. It is easy to use, requires minimal user intervention, can handle multiple sensors and outperforms the state-of-the-art methods in accuracy.

Cameras offer an inexpensive solution for machine vision. They provide high resolution, colorful images at a relatively high frame-rate. Besides that, image processing is a well-studied topic of research, and many algorithms exist already to solve object detection and recognition, camera movement estimation, semantic segmentation, etc. On the other hand, cameras can be used only in appropriate lighting conditions, and problems may occur with occlusion, shadows or in night light.

The LiDAR (Light Detection and Raging) technique applies infrared light to detect objects in its surroundings. 3D LiDARs can map the environment in $360^{\circ }$ and produce a sparse point cloud. These sensors use active illumination; thus, they can also be used in night light. However, they retrieve only low-resolution depth information with an accuracy of 2–5 cm at a maximal refresh rate of 15 Hz. For example, the well-known Velodyne HDL-64 uses 64 vertically-oriented light beams and a rotating head to scan a field of view of $360^{\circ }$. It is a notable issue for this sensor, that 3D scanning is not synchronized due to the rotation: different scannings of the same beam are shifted in time. The compensation of this shift is very challenging when the speed of the vehicle is fast.

Cameras and LiDAR sensors compensate the shortcomings of the other; thus, they can be effectively used jointly in many scenarios. Robotics and autonomous driving are the most popular of these. However, extrinsic calibration is needed for these sensors to effectively work together, meaning that their relative pose needs to be precisely estimated. LiDARs provide sparse point clouds, with only position information, while the cameras provide high resolution color images. The calibration of these different modularities is challenging, yet an important topic.

For calibration, we distinguish online and offline methods. Online calibration means that the sensors are calibrated during the usage of the system, while the latter one indicates that the calibration is done beforehand. Online methods are used when the vehicle cannot be accessed easily for calibration purposes. However, when feasible, offline methods provide more accurate results. Cameras and LiDARs have intrinsic and extrinsic parameters. In this paper, we address the problem of extrinsic, offline calibration. That means that the intrinsic parameters of the sensors are considered to be known a priori, and only the extrinsic parameters need to be calculated, namely the relative rotation and translation of the sensors. It is shown that the calibration can be carried out using an ordinary cardboard box, outperforming the state-of-the-art methods in accuracy, with the need for a single observation of the calibration object.

Rodriguez et al. [1] used a black circle-based planar board to avoid the large noise caused by chessboard patterns. 3D coordinates of the center of the circle and the normal vector of the plane were estimated. Their method needed at least six positions of the calibration object. Finally, the initial guess of the LiDAR-camera rigid transformation was refined by the well-known Levenberg–Marquardt [2,3] (LM) algorithm.

An automatic calibration method was published by Alismail et al. [4]. It used planar calibration object with a black circular region and a marked center. Random Sample Consensus [5] (RANSAC) was applied for plane extraction. The center and normal of the circle were computed based on a single camera view. Finally, point-plane Iterative Closest Point (ICP) [6] was used with nonlinear optimization by LM to refine the extrinsic parameters.

Park et al. [7] used a white, homogeneous, planar triangle or diamond-shaped board for calibration. Several positions were needed from the board or at least three boards at the same time. Another drawback of their algorithm was that the spatial coordinates of the planar board were estimated and not measured. This fact influenced the accuracy of the calibration. The details of this method can be found in Section 6.1.

Gong et al. published a method in [8] that needed at least two scans of the same trihedron object measured by both instruments for the calibration. This produced a significant amount of data to process. In their work, it took 20 s to calibrate using nine observations. The main disadvantage of their method is that the process needed much human intervention, e.g., the separation of the trihedron points and the selection of the related planes in the images needed to be performed manually.

A different type of calibration object was used in Velas et al. [9]. They assumed a planar object containing four circular holes in front of a white background. Their method was based on the work of Levison and Thrun [10]. The holes in both the 3D LiDAR point cloud and the acquired image were detected automatically. However, we cannot apply this method to the point cloud measured by Velodyne VLP-16 LiDAR, due to the sparsity of the acquired point cloud.

Geiger et al. [11] introduced a method to calibrate a LiDAR-camera pair taking only one measurement by the LiDAR and a single image by the camera. The method was fully automatic, however, it needed multiple chessboards and at least two camera images from different positions. The algorithm was briefly introduced in Section 6.1 with a comparison to the proposed method.

Hassanein et al. published a method for the calibration of a stereo camera pair and LiDAR sensor in [12]. Their method required a well-textured calibration object and a pre-calibrated stereo setup. They used Speeded Up Robust Features (SURF) [13] to reconstruct the scene in 3D and ICP [14] to match point clouds of the LiDAR and the stereo reconstruction. The details of this algorithm are discussed in Section 6.1.

Table 1 summarizes the strength and weaknesses of the above-mentioned methods.

The main contributions of the paper are as follows: a new LiDAR-camera calibration is introduced, which uses ordinary cardboard boxes. It achieves high accuracy and can be used with multiple sensors. A new Bundle Adjustment (BA)-based technique is introduced to reduce the overall error of LiDAR-camera system calibration. The method is evaluated on both synthetic and real-world data, competing against state-of-the-art techniques. Moreover, a technique is also presented for estimating the car body border, as a 2D bounding box, with respect to the calibrated sensors.

This study is the extension of our previous paper [15]. The novelty here is the application of BA, comparisons to other methods, the proof for the convergence of the proposed box fitting algorithm and the 2D bounding box calculation.

Our work has limitations, of course: (i) It is an offline calibration approach; therefore, the change in sensor setup during usage cannot be handled. In other words, if the calibration becomes inaccurate due to, e.g., mechanical resonance, the whole calibration procedure has to be repeated; (ii) The calibration needs a special setup that is not always available. However, the accuracy of the proposed calibration algorithms outperforms the state-of-the-art rival techniques, as is shown in this paper.

## 2. Calibration Outline

Offline calibration methods use one or more calibration objects that can be easily detected by both the LiDAR sensor and the camera, as well. Planar calibration objects with a checkerboard pattern [11] or other rich textures are very popular in the literature. A planar board can be easily detected in spatial point clouds, and the pattern of the board is identifiable in the camera image. An example of accurate plane detection can be seen in Section 7, where a technique is presented for 2D bounding box calculation of a car. Using this technique, the accuracy of the bounding box is below two centimeters. However, the texture patterns can cause heavy noise in the LiDAR point cloud, especially the checkerboard pattern [7]. Moreover, all of these methods struggle with the fact that the edges of the board cannot be precisely calculated in a LiDAR point cloud, only the plane itself. The application of a low resolution LiDAR sensor, e.g., the Velodyne VLP-16, is even more challenging. The proposed method is developed for both low and high resolution LiDAR devices; thus, we decided to use a spatial object instead of a planar one.

Cardboard boxes can be found everywhere, and precise ones can be easily manufactured. They have a well-defined shape; therefore, they are a great choice to calibrate cameras and LiDAR device pairs. Their three perpendicular sides can be accurately detected in a LiDAR point cloud. The intersections of the planar sides yield the edges of the box. Corners are precisely calculated, giving the edges and the dimensions of the box. 3D → 2D correspondences are also known, if the projections of these corners are selected in the camera image. In this case, the camera-to-LiDAR calibration is equivalent to the Perspective-n-Point (PnP) problem, which can be solved by several efficient algorithms, e.g., Efficient PnP (EPnP) [16,17].

The calibration is carried out as follows. A cardboard box is placed in the field of view of the LiDAR and camera sensors, in a way that the three perpendicular sides are visible from both of them. The required inputs of the calibration are: (i) camera image(s); (ii) LiDAR point cloud(s) and (iii) length of the box edges.

The proposed method needs only one image per camera and one point cloud per LiDAR sensor. Other calibration methods need multiple observations of the same calibration object to archive the desired accuracy, which makes the calibration procedure time-consuming. The required input of the proposed one is as minimal as possible.

**Figure 1:**
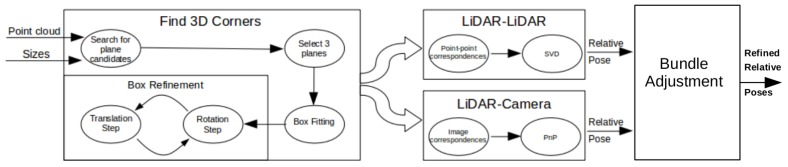
Outline of the calibration. Corners of the calibration box are extracted first from LiDAR point cloud. Then, LiDAR-LiDAR or LiDAR-camera pairs are calibrated using corresponding points.

Figure 1 shows the outline of the calibration procedure. First, coordinates of the box corners are extracted from the point cloud. The rough area of the calibration box needs to be cut manually; however, this cut does not need to be precisely done. Here, the method can robustly find the planes belonging to the calibration box and eliminate other objects falling into this area. After outlier filtering, the intersections of the box planes are calculated. Finally, the corners are refined by an iterative method containing rotations and translations of the fitted box model. The convergence of the iteration is proven, as is discussed in the Appendix. The calibration can be applied to threecases:

1. Camera-LiDAR calibration: Projections of the box corners need to be selected in the camera image. The spatial (LiDAR) and 2D (camera) point correspondences define a PnP problem, which can be effectively solved by, e.g., the EPnP algorithm [16].
2. LiDAR-LiDAR calibration: The corners of the same calibration box need to be calculated in the two point clouds, separately. Then, the extrinsic parameters can be found by point registration.
3. Car Body-LiDAR calibration: The last calibration step is to estimate the car body location with respect to the sensors. This step is independent of the camera-LiDAR and LiDAR-LiDAR calibrations. A single plane is required that can be placed at four different locations: to the left and right side, in front of and behind the car. This step is essential for autonomous driving, as car dimension determines the free space of the car in order to avoid collision.

## 3. Estimation of Box Corners in the LiDAR Point Cloud

The accuracy of the algorithm depends mainly on the accurate calculation of the box corners in the LiDAR point cloud. First, the planes of the box have to be found. It is assumed that three sides of the box are seen both by the camera and the LiDAR sensor.

### 3.1. Finding the Planes of the Box

The rough area of the box needs to be distinguished from the other parts of the point cloud. If no further information about the environment is given, this part is better done manually. One could use retro-reflective material to cover the box and use the reflectivity information of the LiDAR sensors to aid the automatic detection [18,19] of the box. However, our method does not need special materials. As a consequence, the proposed calibration can be used with any type of LiDAR sensor, even if they provide no reflectivity information at all.

After the rough area of the calibration box is cut, the planes of the box are determined. For this purpose, sequential RANSAC [5] is recommended. Sequential RANSAC finds the dominant planes one after the other, maximizing the number of inliers in each iteration, based on their Euclidean distance from the corresponding plane. The threshold, applied for filtering inliers by RANSAC [5], is equal to the measurement accuracy of the LiDAR device, that is 5 cm and 3 cm for the Velodyne HDL-64 and Velodyne VLP-16, respectively. Energy minimization-based methods like PEARL [20] or its improvements [21,22] tend to solve the multi-model fitting better than sequential RANSAC. However, as the number of models cannot be specified, they usually find less than three planes in many cases with sparse point clouds. Thus, we decided not to use them.

**Figure 2:**
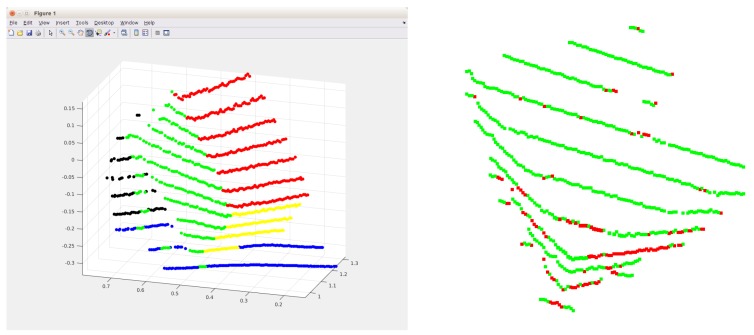
The **left** image shows the result of the sequential RANSAC. Five different planes are found in this case, each marked by different colors. In the **right** image, the result of the outlier filtering can be seen. Green and red points indicate inliers and outliers.

The left image of Figure 2 shows the result of the sequential RANSAC. Five planes have been found in the rough area of the calibration box. The point cloud contains points from another object, a chair, that partly occupies this area. Thus, the perpendicular property of the box is exploited to determine the planes belonging to the calibration box. Three planes that are the most perpendicular to each other are selected from the plane candidates that minimize the following error:

(1) $$E\left(n_{1},n_{2},n_{3}\right)=|n_{1}^{T}n_{2}\left|+\right|n_{1}^{T}n_{3}\left|+\right|n_{2}^{T}n_{3}|,$$

where $n_{k}$ is the normal of the *k*-th plane (k∈{1,2,3}). These normals are computed by Principal Component Analysis (PCA): they are the eigenvectors corresponding to the least eigenvalue of the covariance matrix of the planar point sets. The number of planes is usually low in this area; thus, exhaustive search can be applied to find the planes belonging to the box. In the left image of Figure 2, the red, green and yellow planes are selected as planes that belong to the calibration box. Points of other planes are eliminated.

### 3.2. Outlier Filtering

The LiDAR point cloud can be heavily affected by the intrinsic noise of the LiDAR sensor or the material of the box. Thus, outlier filtering is needed to find and discard noisy points from the point cloud.

RANSAC is used again to remove the outliers. The fitted model consists of three perpendicular planes. Note that in Equation (1), the perpendicularity is not stated. The points are assigned to one of the three planes from the previous step. Points that belong to different planes are denoted as $L_{i}$, where (i∈{1,2,3}). Their ordering does not matter.

The model fitting is as follows: (i) select three random points from $L_{1}$; (ii) select two random points from $L_{2}$; and (iii) select a single random point from $L_{3}$.

First, three points determine a plane in the point cloud. In the second step, two points are chosen to determine the next plane that is perpendicular to the first one. In the final step, only one point is required from $L_{3}$. This point determines the last plane that is perpendicular to the first two ones. Outliers are detected by the minimal Euclidean distance from the planes, using the same threshold as before. An example output of this step can be seen in the right plot of Figure 2, where the green and red points indicate inliers and outliers, respectively.

We remark that the denser point cloud from the Velodyne HDL-64 (equipped with an older sensor) contains more noise, than the sparser one of the Velodyne VLP-16. Outlier filtering becomes even more important for the former sensor, since the next refinement step is based on linear regression.

### 3.3. Iterative Box Refinement

The last step of the algorithm refines the box corners to the filtered point cloud. The outliers are eliminated in the previous step; therefore, the point cloud contains only inlier points. Thus, a least-squares-based refinement can be applied. The cost function contains the sum of all point-plane distances, defined as follows:

(2) $$C=\sum_{i=1}^{3}\sum_{j=1}^{m_{i}}{\left|{\left(p_{j}^{i}-q^{i}\right)}^{T}n^{i}\right|}^{2},$$

where $p_{j}^{1},j\in \left\{1,2,\ldots ,m_{1}\right\}$, $p_{j}^{2},j\in \left\{1,2,\ldots ,m_{2}\right\}$ and $p_{j}^{3},j\in \left\{1,2,\ldots ,m_{3}\right\}$ denote the points in $L_{1}$, $L_{2}$ and $L_{3}$, respectively. $q^{i}$ denotes a point lying on the *i*-th plane, which does not need to be in the set $p_{j}^{i}$, and $n^{i}$ are the normals of the planes.

The refinement contains two steps that are repeated one after the other, until convergence. The first step is the rotation of planes. In this step, a plane pair is rotated around their line of intersection to get the best fit in a least squares manner. In the second step, the planes are translated strictly along their normal vector. These steps preserve the orthogonality of the planes.

#### 3.3.1. Rotation Step

In the rotation step, two planes are chosen and rotated around their line of intersection. The aim of the rotation is to minimize the squared Euclidean distance of the planes from their corresponding points. Let the axis Z be the intersection of the two planes to be rotated. Then, the rotation matrix is as follows:

(3) $$R_{Z}^{T}=\left[\begin{array}{ccc} c & -s & 0\\ s & c & 0\\ 0 & 0 & 1 \end{array}\right],$$

where c=cosγ and s=sinγ.

Without loss of generality, it can be assumed that the points $q^{i}$ represent the single point of intersection of the three planes. The origin of the 3D coordinate system can be selected as that point; thus $q^{1}=q^{2}=q^{3}=0$, and the normal of the three planes is ${\left[1\;0\;0\right]}^{T}$, ${\left[0\;1\;0\right]}^{T}$ and ${\left[0\;0\;1\right]}^{T}$. The rotation does not influence the fitting error of the third plane; therefore, the minimization problem becomes:

(4) $$C^{Rot}=\sum_{i=1,2}\sum_{j=1}^{m_{i}}{\left|{\left(\left[\begin{array}{ccc} c & -s & 0\\ s & c & 0\\ 0 & 0 & 1 \end{array}\right]p_{j}^{i}\right)}^{T}n^{i}\right|}^{2}.$$

After elementary modifications, the optimization problem represented by the above cost is transformed to the minimization of $\left|Ax\right|$ subject to $x^{T}x=1$, where:

(5) $$A=\left[\begin{array}{cc} x_{1}^{1} & -y_{1}^{1}\\ \vdots & \vdots \\ x_{m_{1}}^{1} & -y_{m_{1}}^{1}\\ y_{1}^{2} & x_{1}^{2}\\ \vdots & \vdots \\ y_{m_{2}}^{2} & x_{m_{2}}^{2} \end{array}\right],x=\left[\begin{array}{c} c\\ s \end{array}\right].$$

The optimal angle of rotation is obtained as the eigenvector of matrix $A^{T}A$ corresponding to the smaller eigenvalue (matrix $A^{T}A$ always has two non-negative real eigenvalues). The angle is then calculated as γ=atan2(s,c). The rotation step is repeated for each pair of planes. This closed-form solution can be similarly obtained for the remaining axes X and Y.

#### 3.3.2. Translation Step

After their rotation, the planes are translated strictly along their normal vectors. The aim of the translation is the same as that of the rotation, to obtain the best fit in a least squares manner. The translation of the planes can be expressed as a single vector t of three components. In this case, the cost function is as follows:

(6) $$C^{Trans}=\sum_{i=1}^{3}\sum_{j=1}^{m_{i}}{\left|{\left(p_{j}^{i}-t\right)}^{T}n^{i}\right|}^{2},$$

where t is the optimal translation vector.

This problem can be written as a homogeneous system of linear equations as Bt=c as follows:

(7) $$B=\left[\begin{array}{c} n^{1T}\\ \vdots \\ n^{1T}\\ n^{2T}\\ \vdots \\ n^{2T}\\ n^{3T}\\ \vdots \\ n^{3T} \end{array}\right],\;c=\left[\begin{array}{c} n^{1T}p_{1}^{1}\\ \vdots \\ n^{1T}p_{m_{1}}^{1}\\ n^{2T}p_{1}^{2}\\ \vdots \\ n^{2T}p_{m_{2}}^{2}\\ n^{3T}p_{1}^{3}\\ \vdots \\ n^{3T}p_{m_{3}}^{3} \end{array}\right],$$

where the row vector $n^{\mathrm{jT}}$ denotes the transpose of column vector $n^{j}$. Each term of the cost function gives a line of the equation to the system. The solution is given by the well-known formula $t={\left(B^{T}B\right)}^{-1}B^{T}c$. As the vectors $n^{1}$, $n^{2}$ and $n^{3}$ are perpendicular to each other, matrix B is always non-singular; thus, the matrix inversion can be calculated.

### 3.4. Convergence

The proof of convergence of the iterative refinement can be found in Appendix A. For the initial parameters, we recommend using the parameters of the planes from the outlier filtering step, described in Section 3.2. With these parameters, the algorithm needs usually no more than 20–30 steps until convergence. However, according to our experience, the refinement always converged to the correct minimum, with no initialization.

**Figure 3:**
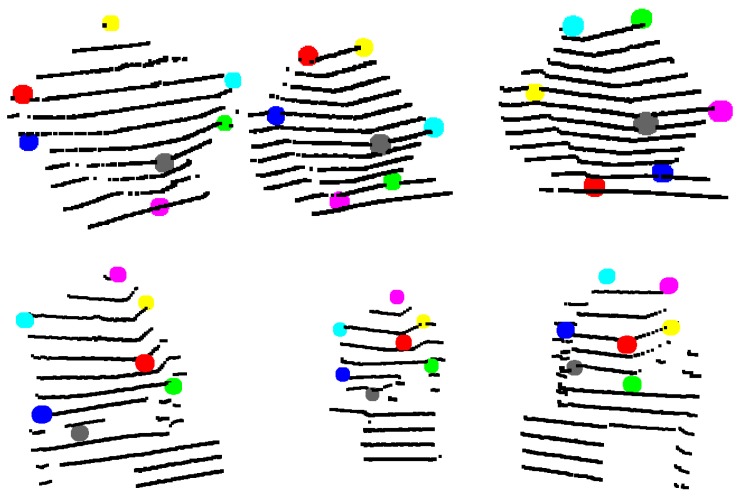
Examples of box corners found in different point clouds. The colored points indicate the calculated box corners, after the refinement.

Figure 3 shows examples of point clouds of boxes. Their calculated corners are represented by different colors. Note that the algorithm can robustly find the box planes and corners. Boxes with different dimensions are tested in various scenarios, where other object jointly occupy the selected neighborhood.

## 4. Getting the Extrinsic Parameters

In the previous section, the calculation of the box corners in a 3D LiDAR point cloud is described. In the following, the extrinsic calibration of devices is divided into two categories. First, the extrinsic calibration of LiDAR-camera pair, then that of a LiDAR-LiDAR pair are described.

### 4.1. LiDAR-Camera Calibration

In the case of the LiDAR and camera calibration, the projections of the 3D corners need to be selected on the camera images. The selected corners are further refined by a corner detector, i.e., the Harris corner detector [23]. The given 3D-2D point correspondences define a PnP problem, which is effectively solved by EPnP [16]. PnP needs at least four correspondences; however, the corners of the calibration box define seven. Thus, it is an over-determined problem even for the case of a single calibration box.

### 4.2. LiDAR-LiDAR Calibration

Suppose that a LiDAR pair is given by their corresponding point clouds. The corners of the calibration box can be found in the two point clouds separately. Then, the extrinsic parameters can be obtained by point registration.

## 5. LiDAR-Camera System Calibration

In the case of multiple LiDAR and camera sensors, a minimization step can be applied, which simultaneously minimizes the overall error of cameras and LiDARs. The proposed method optimizes the parameters of the spatial box(es), as well. The minimization is achieved by two successive steps of numerical refinements of two cost functions: one describing the 3D-to-3D discrepancy of the LiDAR calibration and a re-projection error for the cameras.

The coordinate system of an arbitrarily-selected LiDAR device is set as the reference (origin) of the system; the poses of other cameras ($R_{i}^{C},t_{i}^{C}$), LiDARs ($R_{j}^{L},t_{j}^{L}$) and boxes ($R_{k}^{B},t_{K}^{B}$) are defined w.r.t. it, where indices *i*, *j* and *k* denote the *i*-th camera, *j*-th LiDAR and *k*-th box. The rotation matrix (R) and translation vector (t) define the rigid reference to local coordinate system transformations. The cost functions for a LiDAR-camera pair are as follows:

(8) $$\begin{array}{ccc} {\mathrm{cost}}_{k,j}^{L}\left(P,n\right) & = & {\left|{\left(R_{k}^{B}R_{j}^{L^{T}}\left(P-t_{j}^{L}\right)+t_{k}^{B}\right)}^{T}n\right|}^{2}, \end{array}$$

(9) $$\begin{array}{ccc} {\mathrm{cost}}_{k,i}^{C}\left(Q,q\right) & = & {∥\pi_{i}\left(R_{i}^{C}R_{k}^{B^{T}}\left(Q-t_{k}^{B}\right)+t_{i}^{C}\right)-q∥}_{2}^{2}, \end{array}$$

where the parameter P is a spatial point in the *j*-th LiDAR point cloud, n is a normal vector of the *k*-th observed box. Q and q are the spatial corner point of the *k*-th observed box and the related re-projected coordinate in the *i*-th camera image, respectively. $\pi_{i}$ is the projection function of the *i* camera.

In the case of Equation (8), point P of the *j*-th LiDAR point cloud is transformed to world coordinates using the inverse of the LiDAR pose ($R_{j}^{L},t_{j}^{L}⟶R_{j}^{L^{T}},-R_{j}^{L^{T}}t_{j}^{L}$). Then, world coordinates are easily projected to local coordinates of the *k*-th box using ($R_{k}^{B},t_{K}^{B}$). Having the 3D point measured by the LiDAR now in the local system of the box, the distance w.r.t. the three main planes is computed using the scalar product with the three respective normals, as was introduced before in Equation (2).

In Equation (9), a virtual corner Q of the *k*-th box is mapped to world coordinates using the inverse of ($R_{k}^{B},t_{K}^{B}$). In the next step, the point goes through a world-to-image plane transformation by first applying world-to-camera transformation ($R_{i}^{C},t_{i}^{C}$), then projecting it to the *i*-th image plane by projection function $\pi_{i}$. The computed cost is the squared norm of the difference between the measured point q and the projected one.

The parameters of the the devices, cameras and LiDAR and those of the spatial box(es) are loosely connected; therefore, the Jacobian of the cost function is sparse. In such cases, the BA-paradigm [24] can be applied.

The inputs of this step are (i) the LiDAR point clouds, each point labeled by which box and which face it belongs to; and (ii) the 3D → 2D correspondences between box corners and image points.

In the first pass of the optimization, the LiDAR and box poses are refined numerically: the distance of the LiDAR point cloud to the boxes, represented by three perpendicular planes, is minimized. The minimization is simultaneously carried out in a BA-like manner, refining all LiDAR and box parameters:

(10) $$\min\sum_{\left(k,j,X^{m}\right)\in {\mathrm{Obs}}^{L}}{\mathrm{cost}}_{k,j}^{L}\left(X^{m},n^{m}\right),$$

where ${\mathrm{Obs}}^{L}$ is the set of observations through the LiDARs. Its elements $\left(k,j,X^{m}\right)\in {\mathrm{Obs}}^{L}$ denote point $X^{m}$ of the m∈1,2,3-th side of the *k*-th cube seen by the *j*-th LiDAR. Note that we applied the Huber loss for this case, assuming a noise of 10 cm in case the labeling of the point cloud is not perfect and contains outliers.

The second pass uses the refined and now fixed boxes to refine only the camera poses, based on the following compound cost:

(11) $$\min\sum_{\left(k,i,Q,q\right)\in {\mathrm{Obs}}^{C}}{\mathrm{cost}}_{k,j}^{C}\left(Q,q\right),$$

where ${\mathrm{Obs}}^{C}$ is the set of 3D-to-2D correspondences between cube corners and camera images. An element of this set $\left(k,i,Q,q\right)\in {\mathrm{Obs}}^{C}$ denotes a corner Q of cube *k* observed as image point q on the *i*-th camera.

In the literature, the calibration objects and cameras are usually jointly calibrated and refined [11] in advance of the LiDAR-calibration, to achieve high quality camera and calibration object poses. However, it is essential for such an approach to use multiple cameras, with overlapping fields of view, and/or numerous calibration objects.

The effect of this minimization step on the overall error can be seen in Table 2. It is seen that our BA-like method can significantly reduce the overall calibration cost. The synthetic testing scene consists of two cameras and two LiDARs. Additionally, Gaussian noise is added to the synthetic LiDAR point cloud with 0.04 standard deviation. The real-world test is done using a Velodyne HDL-64, two Velodyne VLP-16 sensors and two RGB cameras.

## 6. Tests

The proposed method is tested both on synthetic and real-world data. The synthetic testing is done by simulating a real-world calibration scenario. The synthetic calibration enables us to compare the calibration methods quantitatively. Real-world calibration is done by low and high resolution LiDAR devices and BlackFly cameras with different optics.

### 6.1. Synthetic Tests

In the synthetic comparison of the algorithms, only a camera and LiDAR pair is considered for calibration; the optimization described in Section 5 is not used. The advantage of these tests is that the exact transformation between the camera and the LiDAR is known; thus, the methods can be compared quantitatively.

For the synthetic tests, the Blender 3D modeling program is used with the Blensor [25] LiDAR simulation package. Blensor is capable of simulating different kinds of LiDAR devices; even their properties can be individually changed.

The synthetic test is done with three different state-of-the-art methods, which are as follows:

1. the KITTI calibration toolbox (denoted as KITTI),
2. the calibration method by Park et al. (denoted as polygonal),
3. the automatic calibration by Hassanein et al. (denoted as multi-camera),
4. and the proposed method (denoted as proposed).

The KITTI Calibration Toolbox (http://www.cvlibs.net/software/calibration/) published by Geiger at al. [11] needs several calibration boards printed with chessboard patterns. The scene has to be observed from at least two camera views and a LiDAR sensor. Multiple chessboards are detected and matched between the images, and the chessboard corners are reconstructed using stereo vision. Then, planes are fitted to the reconstructed and LiDAR point cloud, as well. Finally, these planes are matched. This last step can result in some false calibration results in our scenes; however, it is made more robust by selecting the best scenario with the lowest error.

The rival method, denoted as polygonal was introduced by Park et al. [7]. This calibration uses polygonal (triangle or diamond) white boards for the calibration. The method overcomes the problem of estimating the exact edges of the calibration board by virtual points, which are located between two consecutive LiDAR points, where one is inside and the other is outside the board. From the edges, the corners of the board are calculated, then image points are selected manually, which are then refined by the FAST [26] feature detector. Finally, point correspondences are used for the calibration. In the test, the method shows good results after using four observations of a diamond-shaped board.

Hassanein et al. [12] described a new automatic calibration method based on stereo reconstruction and ICP [14]. Two cameras and a well-textured object are needed for the procedure. The camera system needs to be calibrated a priori; thus, a sparse point cloud can be reconstructed from SURF [13] features. Then, this point cloud is registered to that of the LiDAR sensor by ICP [14]. This method is labeled as multi-camera in the synthetic tests. In the test, the ground truth (*GT*) locations of the sensors are used as the initial parameters for the ICP. Moreover, outlier filtering is done for the matched SURF features using homography fitting. The latter step is needed, otherwise the point cloud reconstructed from camera images contains too many outliers; thus, the result of the ICP is not satisfactory. It must be noted that the authors suggest to use the roughly-estimated manual measurements of the calibration as initial parameters. However, these measurements cannot be made in some cases, e.g., when the LiDARs and cameras are mounted on different positions of a car. Moreover, a camera system calibration is required before the LiDAR-camera system calibration, which takes more time to calibrate, and it could not be done easily for cameras with no shared field of vision.

Table 3 shows the virtual scenes of the calibration methods. The camera images and LiDAR point clouds are also visualized. The point clouds are acquired by Gaussian noise with a standard deviation of 0.02 m. Note that the box of the proposed algorithm is colorized only for better visualization.

**Figure 4:**
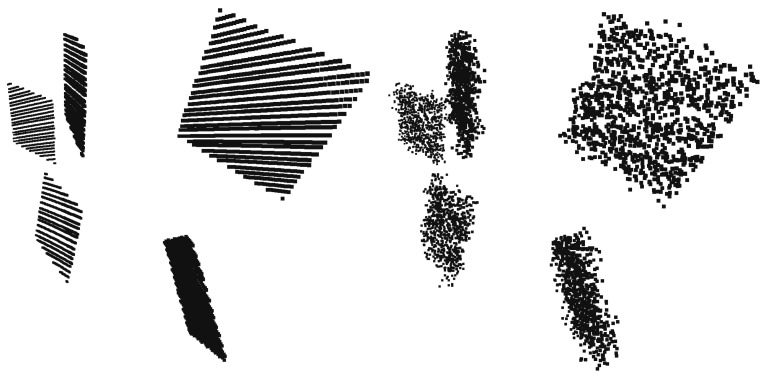
The point cloud of the KITTI virtual calibration scene. **Left** plot: the virtual Velodyne HDL-64 does not contain noise. **Right** plot: Gaussian noise added with zero mean and a 0.14 standard deviation.

The methods are tested against different levels of Gaussian noise, which affects the LiDAR sensor. The noise is added to the distance measured from the sensor; thus, the noisy points are located on the rays cast by the range sensor. The standard deviation and mean values of the Gaussian noise are independently changed. In the first case, the zero-mean Gaussian noise has different levels of the standard deviation between zero and 0.14 m. In the second case, the mean of the Gaussian noise is varied between zero and 0.08 m with a fixed standard deviation of 0.02 m. This latter type of error can be interpreted as systematic noise: point clouds are shifted away from the LiDAR sensor. Figure 4 shows an example of a noise-free (left) and a noisy (right) point cloud of the same scene. The point clouds in the synthetic tests are obtained by simulating a Velodyne-64 sensor.

The extrinsic parameters of the tested calibrations are compared with the *GT*. The parameters consist of a rotation matrix and a translation vector, which represent the transformation between the cameras and the LiDAR. The error of the rotation matrix is compared against the *GT* using the following formula [27]:

(12) $$\alpha =cos^{-1}\left(\left(trace\left(R_{GT}^{T}R\right)-1\right)/2\right),$$

where $R_{GT}$ is the *GT* rotation matrix, retrieved from Blensor data, and R is the rotation matrix obtained by the tested algorithm. We have compared several error metrics, and the characteristics of those were the same. The error of the translation vector is computed as the Euclidean distance between that and the *GT* vector.

**Figure 5:**
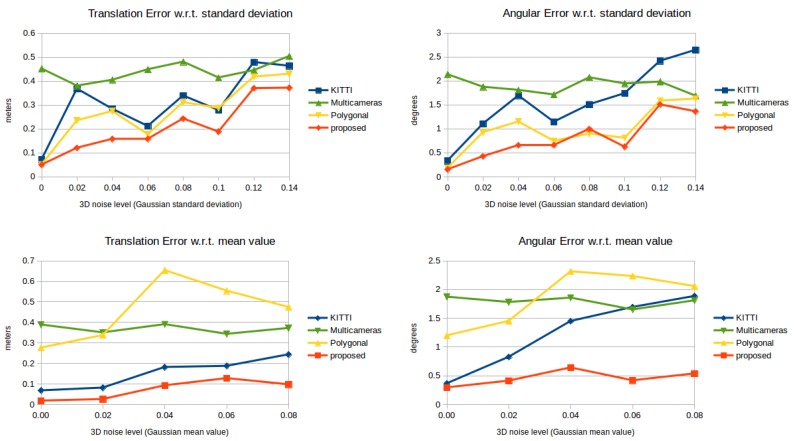
The translation (**left**) and rotation (**right**) errors in the synthetic tests. Top row: the 3D noise level is measured by varying Gaussian standard deviation with zero mean value. Bottom row: varying mean value and a 0.02 standard deviation.

The results of the synthetic test can be seen in Figure 5. In the top row, the translation and angular errors for the tested methods can be seen w.r.t. a zero-mean Gaussian error with a varying standard deviation. The proposed method (red) slightly outperforms the rival methods. The translation error is lower than the rival methods in all cases. The angular error of the polygonal and the proposed one is almost identical, and they perform better than KITTI and multi-camera. This error does not exceed $1.5^{\circ }$ even in the presence of noisy point clouds. In the bottom row of Figure 5, the methods are tested against the varying mean value of the Gaussian noise with a fixed 0.02 standard deviation. The translation errors for the KITTI and the proposed calibration techniques are the lowest. However, the angular error of the former one increases almost linearly w.r.t. the mean value. The angular error of the proposed technique does not exceed $0.6^{\circ }$ even in the case of a 0.08-m mean-valued Gaussian error.

### 6.2. Real-World Tests

Testing these algorithms quantitatively in the real world is challenging, because of the lack of known *GT* transformation. Thus, we choose to re-project some part of the LiDAR point cloud to the image, using the extrinsic parameters of the sensors. If the calibration contains error, then the re-projections of the object will not cover their corresponding pixel locations; thus, the projections are shifted. In the test, a Velodyne VLP-16 LiDAR sensor and two BlackFly (Model BFLY-PGE-13S2C-CS) cameras are applied. The LiDAR sensor detects usually 30,000 points per frame, and the resolution of the camera image is 1288×964 pixels.

**Figure 6:**
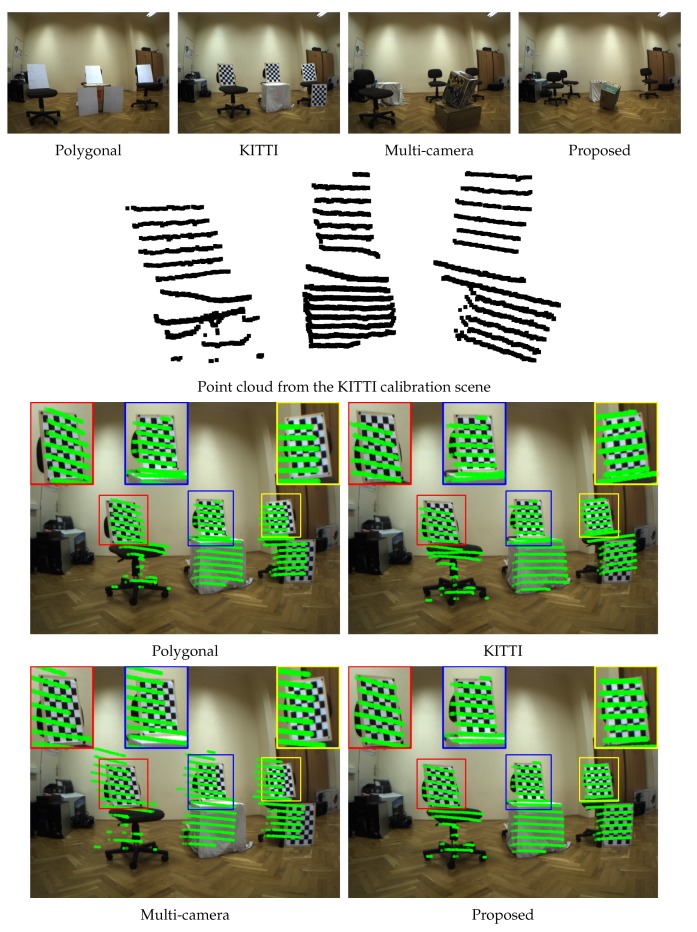
The calibration scenes and re-projections of the methods in the real-world tests. First row: the camera image of the calibration scenes. Second row: the LiDAR point cloud of the foreground objects in the KITTI calibration scene. Bottom row: The re-projections of the previous LiDAR points to the images. The magnified area of the chessboards are shown at the top of the images with the same colors.

The real-world test is carried out as follows. First, two cameras and the LiDAR sensor are fixed on a trunk; therefore, they could not be moved. The calibration scenes for the tested algorithms can be seen in the top row of Figure 6. They are similar to the virtual scenes described in the previous section. The polygonal and KITTI calibrations use boards for the calibration, while the multi-camera and the proposed one use a single box. Note, that the KITTI and the multi-camera methods need two camera images, while the polygonal and the proposed ones need only one for the calibration.

For the KITTI and multi-camera methods, some additional steps need to be done, otherwise the methods fail. For the KITTI calibration, a chessboard is removed from the scene, because it is unable to make correspondences between the five chessboards in the camera images; thus, only four are used. The test is carried out in a room, where many planar objects can be found in the point cloud. The KITTI method is unable to pair the planes coming from the reconstructed point cloud from the cameras and the corresponding planes detected from the LiDAR point cloud. This problem is solved by removing points from the LIDAR point cloud, which are not a part of the chessboards. For the multi-camera method, the pre-calibration of the camera system is done by chessboards, using the widely-used method introduced by Zhang [28]. However, the number of SURF features extracted from the textured box is low, even though it is placed in front of the cameras; see, e.g., the third image of the top row in Figure 6. Thus, three homographies between the box sides are calculated by manually selecting the corners of the box in the images. Then, the point cloud is reconstructed using the SURF features located on the box in the first image via three estimated homographies corresponding to the box sides. These changes make the automatic approaches semi-automatic, but they are needed, otherwise the methods yield unacceptable results. The polygonal and proposed methods work flawlessly in these tests.

After the calibration, the LiDAR points of the foreground objects in the KITTI testing scene are re-projected to the camera image. This point cloud is visualized in the second row of Figure 6. It contains the points of the chairs and chessboards; see the second image of the first row of Figure 6, marked as KITTI. The re-projection is done using the extrinsic parameters of the calibration method, while the intrinsic parameters of the camera are obtained by the calibration of the camera system. In the bottom two rows of Figure 6, the green points mark the re-projected ones. The low quality of a calibration can be recognized if the points are shifted in the image, from the objects they represent in the LiDAR point cloud. If the points in the LiDAR point cloud are re-projected to the correct object locations, then the calibration performs better. In the case of the polygonal and multi-camera methods, the re-projected points of the chessboards are obviously shifted, and this indicates lower quality extrinsic parameters. For the KITTI and proposed methods, the points cover their corresponding object, and this means that these methods perform better. However, note the difference between these two methods by observing the re-projections of the left and right chessboards. The green objects fall off the chessboards for the KITTI calibration, while they cover their corresponding locations using the proposed one. This indicates that our calibration performs better.

**Figure 7:**
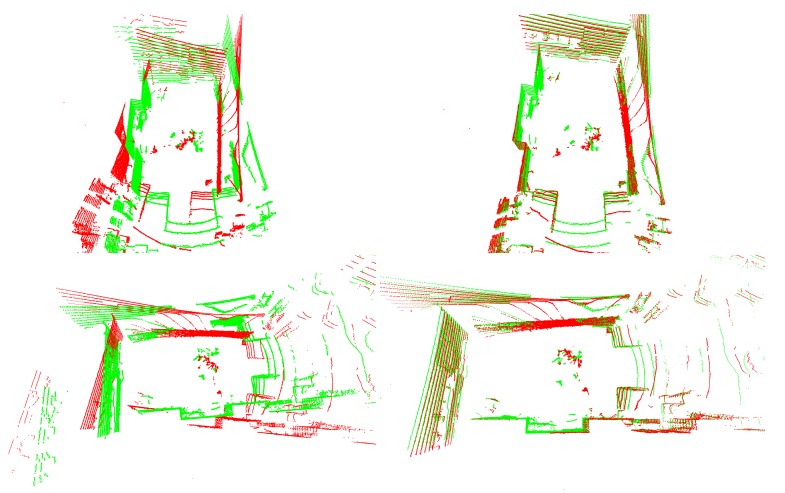
Point cloud fusion of two Velodyne VLP-16 LiDARs. The point clouds are colored in red and green, for the first and second sensors, respectively. **First Column**: without calibration; **Second Column**: with calibration.

**Figure 8:**
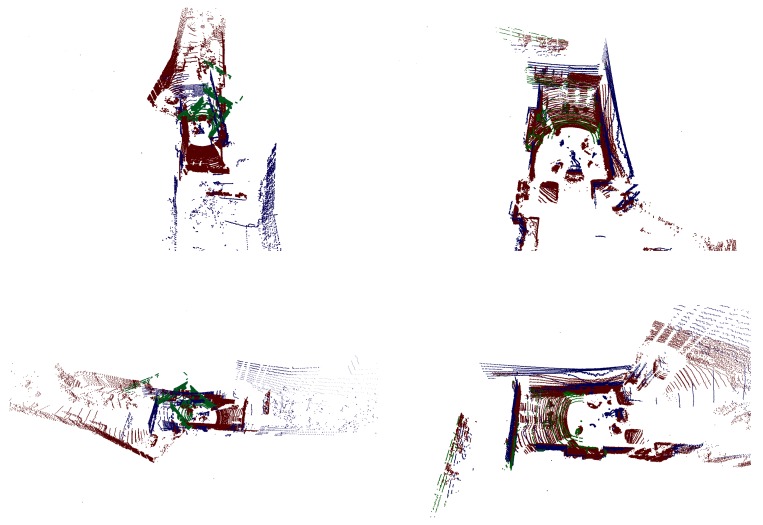

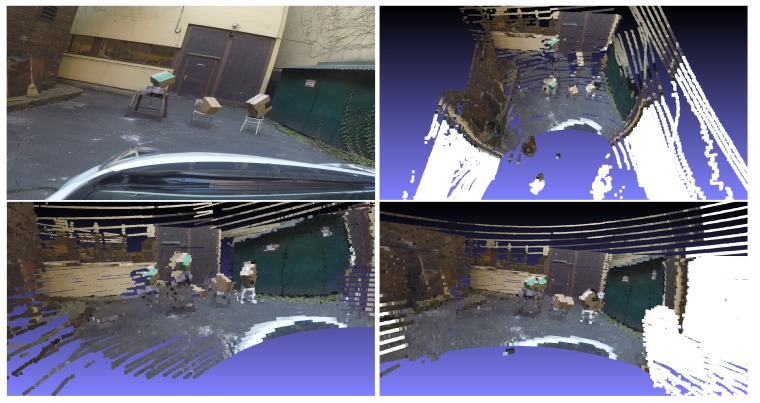
Point cloud fusion of three LiDAR devices, colored by the left camera placed of the top of a car. The LiDAR points from Velodyne HDL-64 and two VLP-16 are colored in red, green and blue points, respectively. The **first column of the top rows** shows the fusion with no calibration, while **the second column** shows with calibration. The **first image in the third row** shows the camera image, and the others are taken from the colored point cloud fusion from the bird’s eye view, the viewpoint of the camera and the Velodyne HDL-64 LiDAR. The occlusion caused by different LiDAR views was not considered during the point cloud coloring process. White points are located outside of the camera view.

Figure 7 shows the result of LiDAR-LiDAR calibration. In this case, two low-resolution point cloud are taken by Velodyne VLP-16 sensors. The point cloud fusions of the first column use no calibration parameters, while the second ones use the extrinsic parameters of the proposed method. Figure 8 shows a point cloud fusion from three LiDARs, a Velodyne HDL-64 and two Velodyne VLP-16. The points from the former LiDAR are colored in red, while that of the latter devices in green and blue colors. The first column of the top rows shows the LiDAR point cloud fusion using no calibration, while in the second column, the parameters of the proposed method are used. The point cloud is colored by projecting the cloud to the camera image using the extrinsic parameters of the calibration and the intrinsic parameters of the camera.

## 7. Calibration of Car Dimensions

It is important to know the size and location/orientation of the car body with respect to the sensor position(s) in the case of autonomous driving. In this scenario, we are interested in a 2D bounding box from a bird’s eye view of the car. For this purpose, a board was used as the calibration object, which was held in parallel with the four sides of the car. The plane of the board was vertical for the sake of simplicity. We show here how the dimensions of a Toyota Prius are calibrated.

However, the Velodyne HDL-64 cannot detect points that are too close to the instrument. Objects that are closer than 1.2–1.5 m are invisible in the point cloud. This is not a problem for measuring the front and back of a Toyota Prius, but it is in case of the sides. During the measurement of the front and near sides, the board was held as near as possible to the car. In the measurement of the left and right sides, the board was moved approximately one meter away, but keeping it aligned. The distance was accurately measured.

**Figure 9:**
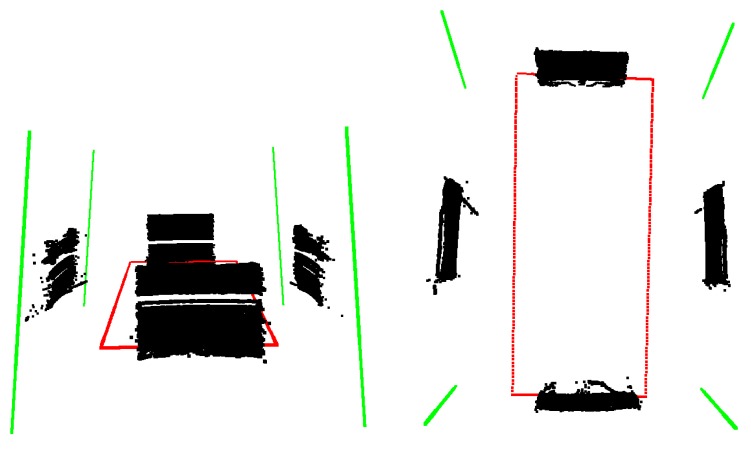
Calibration of the car’s bounding box. The black points are measured by a LiDAR device, and the green and red lines indicate the intersection of the detected planes and the bounding box of the car, respectively.

The black point clouds in Figure 9 indicate the acquired LiDAR points of the four different positions of the board.

After the measurement, the planes are calculated using RANSAC [5], and the detection error of the Velodyne LiDAR is set as a threshold. Then, the intersections of the planes are calculated, and finally, the bounding box is acquired by intersecting the lines with the Z=−0.5 plane. In Figure 9, the green lines mark the intersections of the detected planes, and the calculated bounding box is visualized by red lines.

The size of a Toyota Prius is 4.540 m × 1.760 m according to the official car specification sheet. The dimension of the calculated bounding box is 4.556 m × 1.751 m. Therefore, the error of the bounding box is below two centimeters, which is satisfactory for autonomous driving.

## 8. Conclusions

A novel camera-LiDAR calibration approach is proposed here. It consists of three method: a camera-LiDAR, a LiDAR-LiDAR calibration and a car body size estimation. The camera-LiDAR and LiDAR-LiDAR calibration algorithm use ordinary (cardboard) boxes due to two reasons: (i) the box corners can be determined both in camera images and spatial point clouds; in the latter case, they are the intersections of the box sides; (ii) no special calibration objects are required.

The camera-LiDAR calibration is straightforwardly solved by EPnP algorithm. The most challenging part of the calibration approach is the box detection in LiDAR point clouds. A novel iterative fitting algorithm is introduced here, and its convergence is proven. For multiple sensors, a BA-like minimization technique is introduced in order to refine all the camera and LiDAR parameters.

Finally, a novel technique for 2D bounding box calculation of a car is also presented, based on the fitting of planes in the four principal directions. The dimensions and locations of the car body can be accurately estimated, as well.

## Figures and Tables

**Table 1:** This table summarizes the previous works for LiDAR and camera calibration. Their strengths and weaknesses are also included.

	Automatic	# of Observations	Calibration Object	Strength	Weakness
Rodrigues et al. [1]	no	6	planar with circular hole	low noise ratio by texture	no guarantee for convergence
Alismail et al. [4]	yes	1	black planar circle	automatic	the center of the circle must be marked
Park et al. [7]	no	3	white homogeneous, planar	no additional LiDAR noise by texture	estimated board edges
Gong et al. [8]	no	2	arbitrary trihedron	orthogonality of the object is not required	much human intervention
Velas et al. [9]	yes	1	planar with holes, white background	automatic	difficult calibration setup
Geiger et al. [11]	yes	1	planar, chessboards	only one shot is needed by sensors	multiple chessboards are needed
Hassanein et al. [12]	yes	1	well-textured trihedron	automatic	camera system must be pre-calibrated

**Table 2:** The table shows the root mean square errors of the cameras and LiDARs before and after the Bundle Adjustment (BA)-like technique defined in Section 5. The errors are measured in pixels and meters for the cameras and LiDARs, respectively. The synthetic testing scene consist of two cameras and two LiDARs. The real-world test is done with a Velodyne HDL-64, two Velodyne VLP-16 and two cameras. Additionally Gaussian noise is added to the synthetic LiDAR point cloud with 0.04 standard deviation.

	Before BA		After BA	
	Camera	LiDAR	Camera	LiDAR
Synthetic	4.231 px	0.02231 m	2.152 px	0.02001 m
Real-world	2.892 px	0.03515 m	0.963 px	0.01036 m

**Table 3:** Virtual calibration scenes used for the synthetic tests. Plots from left to right: (i) the 3D scene inside Blensor; (ii) a camera image and (iii) the LiDAR point cloud contaminated by zero-mean Gaussian noise with a 0.02 standard deviation.

Method	3D Scene	Camera	LiDAR
**KITTI**	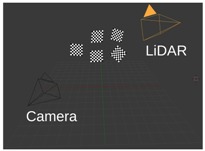	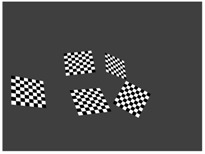	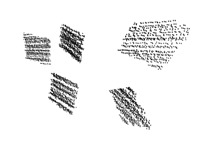
**Polygonal**	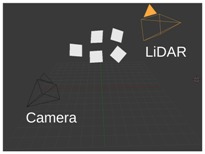	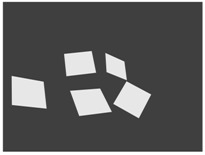	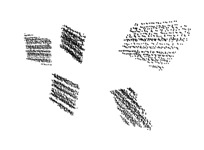
**Multi-camera**	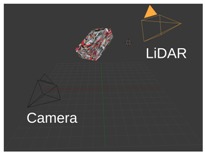	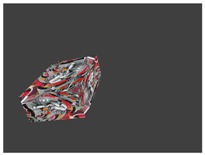	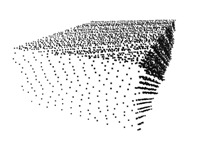
**Proposed**	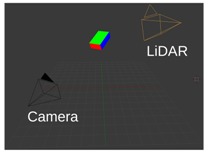	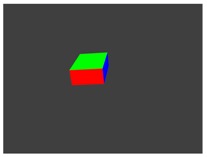	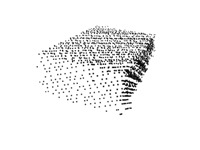

## Appendix A. Convergence of Iterative Box Fitting

It is shown in this section that the iterative refinement method defined in Section 3.3 converges to the closest minimum. As the robust detection selects a good initial value for the parameters, it is probable that the global optimum can be reached. Thus, the initial parameters of the planes do not matter; however, for the sake of low iteration steps, we recommend using the plane parameters from the previous step, the outlier filtering introduced in Section 3.2.

Let us define the least squares cost function as follows:

(A1) $$C=\sum_{i=1}^{3}\sum_{j=1}^{m_{i}}{\left|{\left(p_{j}^{i}-c\right)}^{T}n^{i}\right|}^{2},$$

where $p_{j_{1}}^{1},j_{1}\in \left\{1,2,\ldots ,m_{1}\right\}$, $p_{j_{2}}^{2},j_{2}\in \left\{1,2,\ldots ,m_{2}\right\}$ and $p_{j_{3}}^{3},j_{3}\in \left\{1,2,\ldots ,m_{3}\right\}$ denote the points in $L_{1}$, $L_{2}$ and $L_{3}$, respectively. c denotes the point of intersection of the three planes, and $n^{i}$ are the unit-length normals of the planes. The iterative fitting consists of two steps, a rotation plus a translation.

Rotation: The first step of the iteration is the rotation. This will select two out of three planes and rotate them along their intersection line. For the sake of simplicity, let us assume that the coordinate system is aligned with the intersections of the planes, and the intersection of the planes that will be rotated is the Z axis. The rotation matrix around the axis *Z* was already defined in Equation (3). In this Appendix, we show the effect of the rotation around axis Z on the cost function. The proof is straightforward for the other two rotations around axes X and Y.

The cost function to minimize is as follows:

(A2) $$C^{Rot}=\sum_{i=1}^{3}\sum_{j=1}^{m_{i}}{\left|{\left(R_{Z}^{T}p_{j}^{i}-c\right)}^{T}n^{i}\right|}^{2},$$

where the center of intersection is the origin and is zero $c={\left[0,0,0\right]}^{T}$ and the normals ($n^{i}$) are ${\left[1,0,0\right]}^{T}$, ${\left[0,1,0\right]}^{T}$, ${\left[0,0,1\right]}^{T}$, respectively. The cost function can be modified as:

(A3) $$C^{Rot}=\sum_{j=1}^{m_{1}}{\left|{p_{j}^{1}}^{T}R_{Z}{\left[1,0,0\right]}^{T}\right|}^{2}+\sum_{j=1}^{m_{2}}{\left|{p_{j}^{2}}^{T}R_{Z}{\left[0,1,0\right]}^{T}\right|}^{2}+\sum_{j=1}^{m_{3}}{\left|{p_{j}^{3}}^{T}R_{Z}{\left[0,0,1\right]}^{T}\right|}^{2}.$$

Note, that the rotation $R_{Z}$ does not affect the cost of the third plane:

(A4) $$C^{Rot}=\sum_{j=1}^{m_{1}}{\left|{p_{j}^{1}}^{T}R_{Z}{\left[1,0,0\right]}^{T}\right|}^{2}+\sum_{j=1}^{m_{2}}{\left|{p_{j}^{2}}^{T}R_{Z}{\left[0,1,0\right]}^{T}\right|}^{2}+\sum_{j=1}^{m_{3}}{\left|z_{j}^{3}\right|}^{2},$$

where $z_{j}^{3}$ denotes the *z* coordinate of a point $p_{j}^{3}$. Since the rotation angle (γ) can be obtained as described in Section 3.3, Equation (A4) is minimized using $R_{Z}$; thus, *C* is minimized as well.

Translation: As the error vector is linear with respect to the translation error t as is written in Equation (7), it can be optimally solved in the least-squares step.

Convergence: As both the rotation and translation steps decrease the same norm in the least squares sense, the non-negative error cannot be increased within the iteration, and the algorithm can reach the infinitely close local minimum.

## References

[1] Fremont V., Bonnifait P. Extrinsic calibration between a multi-layer lidar and a camera. Proceedings of the 2008 IEEE International Conference on Multisensor Fusion and Integration for Intelligent Systems.

[2] Levenberg K. (1944). A method for the solution of certain problems in least squares. Q. Appl. Math..

[3] Marquardt D. (1963). An algorithm for least-squares estimation of nonlinear parameters. SIAM J. Appl. Math..

[4] Alismail H., Baker L.D., Browning B. Automatic calibration of a range sensor and camera system. Proceedings of the 2012 Second International Conference on 3D Imaging, Modeling, Processing, Visualization & Transmission.

[5] Fischler M.A., Bolles R.C. (1981). Random Sample Consensus: A Paradigm for Model Fitting with Applications to Image Analysis and Automated Cartography. Commun. ACM.

[6] Chen Y., Medioni G. (1992). Object Modelling by Registration of Multiple Range Images. Image Vis. Comput..

[7] Park Y., Yun S., Won C.S., Cho K., Um K., Sim S. (2014). Calibration between color camera and 3D LIDAR instruments with a polygonal planar board. Sensors.

[8] Gong X., Lin Y., Liu J. (2013). 3D LIDAR-Camera Extrinsic Calibration Using an Arbitrary Trihedron. Sensors.

[9] Veĺas M., Španěl M., Materna Z., Herout A. (2014). Calibration of RGB Camera With Velodyne LiDAR. WSCG 2014 Communication Papers Proceedings.

[10] Levinson J., Thrun S. Automatic Online Calibration of Cameras and Lasers. Proceedings of the Robotics: Science and Systems.

[11] Geiger A., Moosmann F., Car O., Schuster B. Automatic camera and range sensor calibration using a single shot. Proceedings of the 2012 IEEE International Conference on Robotics and Automation (ICRA).

[12] Hassanein M., Moussa A., El-Sheimy N. (2016). A new automatic system calibration of multi-cameras and lidar sensors. ISPRS Int. Arch. Photogramm. Remote Sens. Spat. Inf. Sci..

[13] Bay H., Ess A., Tuytelaars T., Van Gool L. (2008). Speeded-Up Robust Features (SURF). Comput. Vis. Image Underst..

[14] Besl P.J., McKay N.D. (1992). A Method for Registration of 3-D Shapes. IEEE Trans. Pattern Anal. Mach. Intell..

[15] Pusztai Z., Hajder L. Accurate Calibration of LiDAR-Camera Systems Using Ordinary Boxes. Proceedings of the 2017 IEEE International Conference on Computer Vision Workshops.

[16] Lepetit V., Moreno-Noguer F., Fua P. (2009). EPnP: An Accurate O(n) Solution to the PnP Problem. Int. J. Comput. Vis..

[17] Zheng Y., Kuang Y., Sugimoto S., Åström K., Okutomi M. Revisiting the PnP Problem: A Fast, General and Optimal Solution. Proceedings of the 2013 IEEE International Conference on Computer Vision.

[18] Pandey G., McBride J.R., Savarese S., Eustice R.M. (2015). Automatic Extrinsic Calibration of Vision and LiDAR by Maximizing Mutual Information. J. Field Robot..

[19] Wang W., Sakurada K., Kawaguchi N. (2017). Reflectance Intensity Assisted Automatic and Accurate Extrinsic Calibration of 3D LiDAR and Panoramic Camera Using a Printed Chessboard. Remote Sens..

[20] Isack H., Boykov Y. (2012). Energy-Based Geometric Multi-model Fitting. Int. J. Comput. Vis..

[21] Pham T., Chin T., Schindler K., Suter D. (2014). Interacting Geometric Priors For Robust Multimodel Fitting. IEEE Trans. Image Process..

[22] Toldo R., Fusiello A., Forsyth D., Torr P., Zisserman A. (2008). Robust Multiple Structures Estimation with J-Linkage. Computer Vision—ECCV 2008.

[23] Harris C., Stephens M. A combined corner and edge detector. Proceedings of the Fourth Alvey Vision Conference.

[24] Triggs B., McLauchlan P.F., Hartley R.I., Fitzgibbon A.W., Triggs W., Zisserman A., Szeliski R. (2000). Bundle Adjustment—A Modern Synthesis. Vision Algorithms: Theory and Practice.

[25] Blender Sensor Simulation. http://www.blensor.org.

[26] Rosten E., Drummond T. Fusing Points and Lines for High Performance Tracking. Proceedings of the Internation Conference on Computer Vision.

[27] Eberly D. (2002). Rotation Representations and Performance Issues.

[28] Zhang Z. (2000). A flexible new technique for camera calibration. IEEE Trans. Pattern Anal. Mach. Intell..

